# Scientific Validation of the Medicinal Efficacy of *Tinospora cordifolia*

**DOI:** 10.1155/2013/292934

**Published:** 2013-12-23

**Authors:** Amita Mishra, Shashank Kumar, Abhay K. Pandey

**Affiliations:** Department of Biochemistry, University of Allahabad, Allahabad 211002, India

## Abstract

Present communication reports the scientific evaluation of *Tinospora cordifolia* for its medicinal efficacy which includes phytochemical screening, antimicrobial, antioxidant, and anticancer activities of the plant. Secondary metabolites including anthraquinones, terpenoids, and saponins were present in many extracts in addition to phenolics. Total phenol contents in various extracts were found in the range of 8.75–52.50 catechol equivalent per gram (CE/g). In disc diffusion assays, polar extracts exhibited considerable inhibition against *Klebsiella pneumoniae*. Several other extracts also showed antibacterial activity against pathogenic strains of *E. coli*, *Pseudomonas* spp., and *Proteus* spp. Minimum bactericidal concentration (MBC) values of potential extracts were found between 1.29 and 22.73 mg/mL. The lowest MBC (1.29 mg/mL) was recorded for acetone and ethyl acetate extracts against *K. pneumoniae* and *Pseudomonas* spp., respectively. The antioxidant activity of the extracts was comparable to that of standard antioxidants and concentration-dependent response was shown in reducing power assay. Aqueous extracts demonstrated substantial metal ion chelating activity (67–95%) at lower concentrations (10–40 **μ**g/mL). Other extracts also exhibited considerable metal chelating response. Most of the extracts revealed considerable inhibition of MCF-7 cancer cell line. The study established remarkable antibacterial, antioxidant, and anticancer potential in *T. cordifolia* stem extracts.

## 1. Introduction

Natural products have been traditionally accepted as remedies for many diseases. The beneficial medicinal effects of plant products typically result from the combinations of secondary metabolites present in the plants. The most important of these bioactive constituents are phenolics, flavonoids, alkaloids, and tannins [1]. Plant extracts have been known since antiquity to possess notable biological activities, including antibacterial, antioxidant, and anticancer properties. It is popular belief that they present minor side effects. Infectious diseases are the leading cause of death worldwide. The ever increasing resistance of pathogens to antibiotics as well as the undesirable side effects of certain antimicrobial agents has necessitated the discovery of novel bioactive compounds [2]. There has been an increasing interest in medicinal plants as a natural alternative to synthetic drugs. Several members of enterobacteriaceae are responsible for causing severe infections. Many reports have been published in recent years on the antimicrobial activity of essential oils and crude extracts derived from plants against etiological agents of infectious diseases and food-borne pathogens [3, 4].

Excessive free radical production in the body leads to a condition known as oxidative stress which produces degenerative effects on human health, resulting in oxidative deterioration of lipids, proteins, and DNA, activation of procarcinogens, inhibition of cellular and antioxidant defense systems, and changes in gene expression and contributing significantly to human disease [5]. Antioxidants have been shown to prevent oxidative damage caused by free radicals and are constantly required to maintain an adequate level of oxidants in order to balance the reactive oxygen species (ROS) in human body. Phytochemicals have capability to protect against ROS-mediated damage and thus have potential application in prevention and curing of diseases [6]. Natural antioxidants such as flavonoids, tannins, coumarins, curcuminoids, xanthones, phenolics, and terpenoids are found in various plant products such as fruits, leaves, seeds, and oils [7].

Cancer is a class of diseases in which a group of cells display uncontrolled growth, invasion, and someti1mes metastasis. Cancer may affect people at all ages, even fetuses, but the risk for most varieties increases with age. Cancer causes about 13% of all human deaths. According to the American Cancer Society, 7.6 million people died from cancer in the world during 2007 [8]. Secondary metabolites are potential anticancer drugs as they may cause either direct cytotoxicity on cancer cells or may affect processes involved in tumor development [9].

*Tinospora cordifolia* (Menispermaceae) is an herbaceous vine indigenous to the tropical areas of India, Myanmar, and Sri Lanka. In vernacular, it is known as amrita, guduchi, shindilkodi, giloy, and so forth. It is widely used in indigenous systems of medicine [10, 11]. The aqueous extract of *T. cordifolia* stem has shown to produce immunological activity due to the presence of arabinogalactan. The plant is known for its antispasmodic, antipyretic, antineoplastic, hypolipidemic, hypoglycemic, immunopotentiating, and hepatoprotective properties. It is also used in general debility, digestive disturbances, loss of appetite and fever in children, dysentery, gonorrhoea, urinary diseases, viral hepatitis, and anaemia [12–14]. Present communication reports the scientific evaluation of medicinal efficacy of *T. cordifolia* as antibacterial, antioxidant, and anticancer agents.

## 2. Materials and Methods

### 2.1. Plant Material and Preparation of Extracts

The *T. cordifolia* stem was shade-dried, crushed, and ground into fine powder with mortar and pestle. Powdered material was sequentially extracted with petroleum ether (PE), benzene (BZ), chloroform (CH), ethyl acetate (EA), acetone (AC), ethyl alcohol (ET), and water (AQ) in Soxhlet apparatus as described earlier [2, 7]. The respective extract fractions were centrifuged, filtered, and lyophilized. The dried residues were dissolved in DMSO for determination of antibacterial, antioxidant, and anticancer activities.

### 2.2. Phytochemical Screening

Phytochemical screening of *T. cordifolia* stem extracts was preformed for the qualitative detection of reducing sugars, anthraquinones, terpenoids, phenolics, flavonoids, saponins, tannins, alkaloids, and cardiac glycosides using standard procedures [2, 15, 16].

### 2.3. Determination of Total Phenolics

Total phenolic content in extract fractions was determined according to the protocol [17, 18] with some modifications [19]. Modifications included dissolution of extracts in DMSO instead of water. 0.2 mL of sample (2 mg/mL in DMSO) was diluted to 3 mL with water. Small amount (0.5 mL) of twofold diluted FCR was added and the contents were mixed. After 3 min, 2 mL of 20% sodium carbonate solution was added and the tubes were placed in boiling water bath for one min followed by cooling. The absorbance was measured at 650 nm against a reagent blank using spectrophotometer (Visiscan 167, Systronics). The concentration of phenols in the test samples was expressed as mg catechol equivalent per gram (mg CE/g). The estimation was performed in triplicate, and the results were expressed as mean ± SEM.

### 2.4. Microorganisms and Growth Conditions

Pathogenic bacteria used in the study were obtained from the Clinical Microbiology Laboratory, Department of Microbiology, MLN Medical College, Allahabad, India. These included Gram negative bacteria (*Escherichia coli, Klebsiella pneumonia, Pseudomonas aeruginosa*, and *Proteus *spp.). The bacterial culture was maintained at 4°C on nutrient agar slants.

### 2.5. Evaluation of Antimicrobial Activity

Antimicrobial activity of plant extracts was determined using Kirby-Bauer disc diffusion method [20]. The inoculum suspension of bacterial strains was swabbed on the entire surface of Mueller-Hinton agar (MHA). Sterile 6 mm diameter paper discs (Himedia) saturated with 20 *μ*L of extracts prepared in DMSO (containing 3.33 to 10 mg extract/disc) were aseptically placed on the upper layer of the inoculated MHA surfaces and plates were incubated at 37°C for 24 hours. Antibacterial activity was determined by measuring diameter of the zone of inhibition (ZOI) surrounding discs. Standard antibiotic discs meropenem (10 *μ*g/disc) and piperacillin tazobactam (100/10 *μ*g/disc) were used as positive controls. Discs containing 20 *μ*L DMSO were used as a negative control. Antimicrobial assay was performed in triplicate and results are reported as mean ± standard deviation of three replicates.

### 2.6. Determination of Minimum Bactericidal Concentration (MBC)

The MBC of the stem extracts was determined using the broth dilution technique [21, 22]. Stock solution (500 mg/mL) of test extracts was prepared. Several tubes containing decreasing dilution of extracts in broth were inoculated with 100 *μ*L of standardized bacterial suspension (10^8^ CFU/mL, 0.5 McFarland standard). The concentration of samples in tubes varied from 227.3 mg/mL to 0.15 mg/mL. All the tubes were incubated overnight at 37°C in BOD incubator. The lowest concentration which did not show any growth of test organism after macroscopic evaluation is defined as minimum inhibitory concentration (MIC). Since most of the tubes containing extracts were coloured, it was difficult to evaluate them for MIC. Therefore, MBC was determined by subculturing the contents on solid agar media. A Loopful of the content of each test tube was inoculated by streaking on a solidified MacConkey agar plate and then incubated at 37°C for 24 hours for possible bacterial growth. The lowest concentration of the extract in subculture that did not show any bacterial growth on plates was considered the MBC.

### 2.7. Reducing Power Assay

The reducing power of test extracts of *T. cordifolia *stem was determined by the methods of Oyaizu [23] with slight modifications [24]. One mL aliquots of extracts (0.66–3.33 mg/mL) prepared in DMSO was taken in test tubes. To each test tube 2.5 mL of phosphate buffer (0.2 M, pH 6.6) and 2.5 mL of 1% potassium hexacyanoferrate (K_3_Fe (CN)_6_) were added and contents were mixed. Tubes were incubated at 50°C in a water bath for 20 min. The reaction was stopped by adding 2.5 mL of 10% TCA and then centrifuged at 4000 g for 10 min. One mL of the supernatant was mixed with 1 mL of distilled water and 0.5 mL of FeCl_3_ solution (0.1%, w/v) and kept at 25°C for 2 min. The reaction led to formation of greenish blue colour. The absorbance was measured at 700 nm. All the tests were run in triplicate and results are reported as mean ± SD. Increase in absorbance of the reaction indicated the higher reducing power of the test samples.

### 2.8. Metal Ion Chelating Activity

The chelation of ferrous ions by the *T. cordifolia *stem extracts was estimated by the method of Dinis et al. [25] as modified by us [6]. Briefly, samples (200 *μ*L) containing 10–40 *μ*g extracts were prepared in DMSO and the volume was raised to 1 mL with methanol. Further 3.7 mL methanol followed by 50 *μ*L of FeCl_2_ (2 mM) was added. The reaction was initiated by the addition of 5 mM ferrozine (0.2 mL) and the mixture was shaken vigorously and left standing at room temperature for 10 min. Absorbance of the pink violet solution was then measured spectrophotometrically (Elico UV-Vis SL 164) at 562 nm. The inhibition percentage of ferrozine-Fe^2+^ complex formation was calculated by the formula given below:

(1) $$\begin{array}{c} \text{\% metal ion chelating ability}\;=\left[\frac{\left(A_{0}-A_{1}\right)}{A_{0}}\right]\times 100, \end{array}$$

where *A*_0_ is the absorbance of control and *A*_1_ is absorbance in the presence of the sample/standard compounds. The results were expressed as mean ± SD of three replicates.

### 2.9. Cell Lines, Growth Conditions, and Treatment

Human cancer cell lines, namely, prostrate (DU-145), ovary (IGR-OV-1), and breast (MCF-7) cell lines were procured from the National Center for Cell Sciences, Pune, India. Cell lines were grown and maintained in RPMI-1640 medium, pH 7.4 with 10% FCS, 100 units/mL penicillin, 100 *μ*g/mL streptomycin, and 2 mM glutamine. Cells were grown in CO_2_ incubator (Heraeus, GmbH Germany) at 37°C in the presence of 90% humidity and 5% CO_2_.

### 2.10. Cytotoxic Assay by Sulforhodamine B Dye (SRB Assay)

The *in vitro *cytotoxicity of stem extracts was determined using sulforhodamine B (SRB) assay [26]. Cell suspension (100 *μ*L, 1 × 10^5^ to 2 × 10^5^ cells per mL depending upon mass doubling time of cells) was grown in 96-well tissue culture plate and incubated for 24 hours. 100 *μ*L test extract (100 *μ*g/well) was then added to the wells and cells were further incubated for another 48 h. The cell growth was arrested by layering 50 *μ*L of 50% TCA, incubated at 4°C for an hour followed by washing with distilled water, and then air-dried. SRB (100 *μ*L, 0.4% in 1% acetic acid) was added to each well and plates were incubated at room temperature for 30 min. The unbound SRB dye was washed with 1% acetic acid and then plates were air-dried. Tris-HCl buffer (100 *μ*L, 0.01 M, pH 10.4) was added and the absorbance was recorded on ELISA reader at 540 nm. Suitable blanks and positive controls were also included. Each test was done in triplicate. The values reported here are mean ± SD of three experiments.

### 2.11. Statistical Analysis

All experiments were carried out in triplicate and data were expressed as mean ± standard deviation (SD) or standard error of mean (SEM). The plots were prepared using Microsoft Excel and Graph Pad Prism software. Data were analyzed using one-way ANOVA.

## 3. Results

### 3.1. Phytochemical Screening of *T. cordifolia* Extracts

All the extracts tested positive for anthraquinones, terpenoids, and phenols (Table 1). Reducing sugar was found in EA, saponin in AQ, alkaloids in PE and ET, and cardiac glycosides in AC and ET extracts. Polar extracts (EA, AC, ET, and AQ) were found positive for tannins.

### 3.2. Total Phenol Contents in Samples

Results have been reported in mg catechol equivalent per gram (mg CE/g) sample (Table 2). Differential content of phenolics (8.75–52.5 mg CE/g) was present in all the extracts. Polar fractions exhibited better extractability of phenolics as shown in Table 2.

### 3.3. Antibacterial Activity of *T. cordifolia*

*T. cordifolia *extracts exhibited variable inhibitory response against pathogenic bacteria. *Pseudomonas* spp. was sensitive to most of the extracts (Table 3). *Proteus* spp. exhibited resistance to most of the extracts. Only polar fractions (AC, ET, and AQ) produced moderate inhibition against *K. pneumoniae* with ZOI ranging from 10.33 mm to 12.33 mm. *E. coli* exhibited appreciable sensitivity to EA and AC extracts with ZOI values of 26.33 mm and 19 mm, respectively, at 10 mg/disc. Merpenem accounted for 37 mm inhibition zone. Similarly, EA and AC fractions also showed significant inhibition potential against *Pseudomonas* spp. (ZOI 17.67 mm and 14.67 mm, resp.). Lower activity was recorded in PE, CH, and ET extracts against *Pseudomonas* spp.

### 3.4. Minimum Bactericidal Concentration (MBC) of *T. cordifolia* Stem Extracts

MBC was determined for potent extracts by the broth microdilution technique. Stock solutions of these potential extracts were serially diluted to produce a number of tubes having final extract concentration in the range of 227.3 mg/mL to 0.15 mg/mL. MIC values could not be determined because broth cultures containing most of the test extracts were coloured in appearance. So it was not possible to observe bacterial turbidity appropriately. Therefore, MBC was determined from MIC tubes. MBC was recorded as the highest dilution of extract in broth samples showing complete absence of growth on agar plates after subculturing on MacConkey's agar for Gram −ve bacteria. Minimum bactericidal concentration (MBC) for effective extracts against pathogenic bacteria was found in the range of 1.29–22.73 mg/mL (Table 4).

### 3.5. Reducing Power Assay

**Figure 1:**
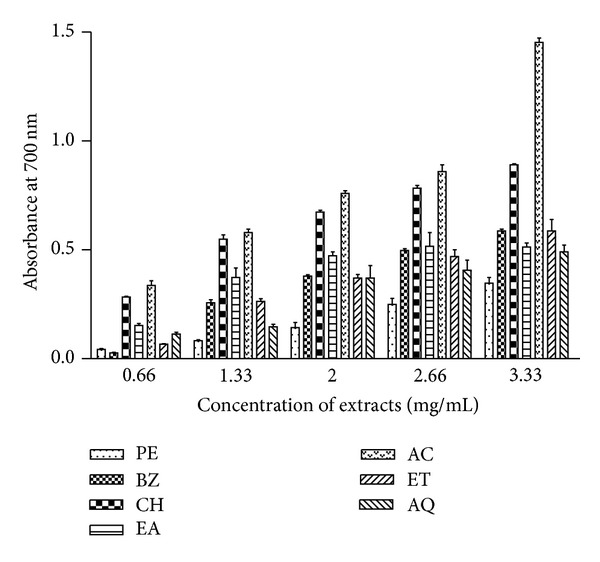
Reducing power of* T. cordifolia *stem extracts. Phytochemicals present in sample were extracted with petroleum ether (PE), benzene (BZ), chloroform (CH), ethyl acetate (EA), acetone (AC), ethanol (ET) and water (AQ) as described in Section 2. Reducing power of extracts was measured at different concentrations (0.66–3.33 mg/mL) and absorbance was recorded at 700 nm. The results are expressed as mean ± SD of three replicates (*P* < 0.05).

Reducing power of extracts was determined at five concentrations (0.66, 1.33, 2.0, 2.66, and 3.33 mg/mL) and the results of reductive efficacy of extracts are depicted in Figure 1. It was observed that the reducing power increased with increasing concentration of extracts. Some of the nonpolar fractions showed comparatively better reducing power. Significant activities were also recorded in CH and AC extracts. The rest of the extracts displayed lower reducing power.

### 3.6. Metal Ion Chelating Activity of Extracts

**Figure 2:**
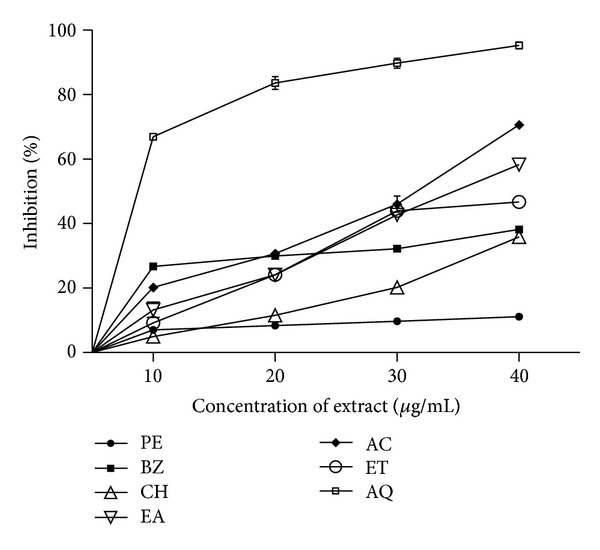
Metal ion chelating activity of* T. cordifolia* stem extracts. Phytochemicals present in sample were extracted with petroleum ether (PE), benzene (BZ), chloroform (CH), ethyl acetate (EA), acetone (AC), ethanol (ET), and water (AQ) as described in Section 2. Metal ion chelating activity of extracts was measured at different concentrations (10–40 *μ*g/mL) and absorbance was recorded at 562 nm. The results are expressed as mean ± SD of three replicates (*P* < 0.05).

The chelating activity was measured at four different concentrations (10, 20, 30, and 40 *μ*g/mL) of the extracts and standard antioxidants. Some of the *T. cordifolia *extracts exhibited potential chelating activity (Figure 2). The metal chelating activity increased with increasing concentration of extracts. AQ extracts demonstrated appreciable chelating activity. Extracts at lower test concentration (10 *μ*g/mL) produced very low chelating power (5–26%) except AQ (67%). The percent chelating activity of AQ extract in the concentration range 10–40 *μ*g/mL was found to be 67–95%. Metal chelating capacity for BHA and BHT (not shown in figure) at test concentrations was comparatively low (44–55%). Nonpolar fractions exhibited low activity (11–38%). The order of antioxidant activity of extracts of *T. cordifolia *was recorded as AQ, AC, EA, ET, BZ, CH, and PE.

### 3.7. Cytotoxic Activity

**Figure 3:**
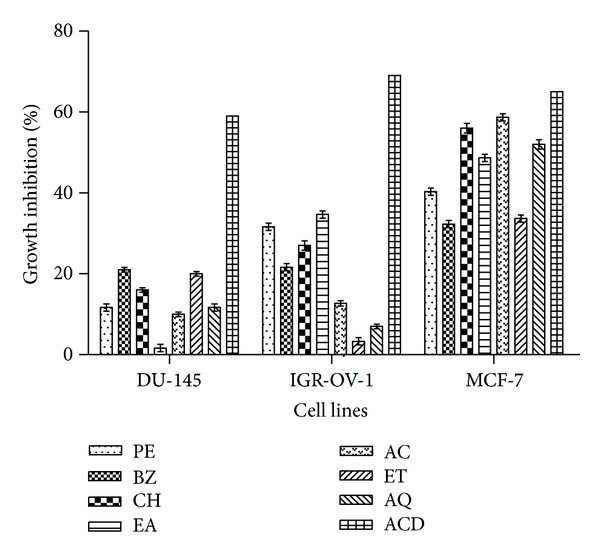
Cytotoxic effect of *T. cordifolia* extracts against human cancer cell lines. Percentage growth inhibition of cell line was assayed at 100 *μ*g/mL concentration of extracts using SRB assay as described in Section 2. PE: petroleum ether, BZ: benzene, CH: chloroform, EA: ethyl acetate, AC: acetone, ET: ethanol, and AQ: water. ACD: Anticancer drugs (mitomycin-C (10 *μ*M) against prostate (DU-145), paclitaxel (10 *μ*M) against lung (HOP-62) and breast (MCF-7), adriamycin (1 *μ*M) against ovary (IGR-OV-1), and 5-fluorouracil (20 *μ*M) against leukemia (THP-1) human cancer cell lines). Data represent mean ± SD of three replicates (*P* < 0.05).

The *in vitro* cytotoxic effect of seven extracts (PE, BZ, CH, EA, AC, ET, and AQ) derived from *T. cordifolia* were evaluated on three human cancer cell lines from different tissues of origin, namely, ovary (IGR-OV-1), prostrate (DU-145), and breast (MCF-7) cancer cell lines. The cytotoxic activity of extracts was compared with the activity of standard anticancer drugs. *T. cordifolia* extracts exhibited moderate cytotoxic potential (Figure 3). CH, AC, and AQ extracts demonstrated cytotoxic activity against MCF-7 cell line with 52–59% growth inhibition. The rest of the extracts produced 34–49% growth inhibition against breast cancer cells. The growth inhibition responses of extracts against IGR-OV-1 and DU-145 cell lines were less than 36%. Several standard anticancer drugs were used as positive control for comparison. The drugs included mitomycin-C (10 *μ*M) against prostate (DU-145), paclitaxel (10 *μ*M) against breast (MCF-7), and adriamycin (1 *μ*M) against ovary (IGR-OV-1) cancer cell lines. Percent growth inhibitions resulting from drugs on different cell lines used in the study were found between 59 and 69%. Cytotoxic effect of some extracts was comparable to that of standard drugs.

## 4. Discussion

Phytochemical screening of the *T. cordifolia *revealed presence of some of the phytoconstituents in all the extracts such as phenols, anthraquinones, and terpenoids (Table 1). Chemical basis of their presence in different fractions may be correlated with small structural differences in the compounds belonging to same group that are critical to their activity as well as solubility. Occasionally tannins and terpenoids will be found in the aqueous phase, but they are more often obtained by treatment with less polar solvents [27]. Since phenols have been attributed with antimicrobial and free radical scavenging activities, they were quantified. Higher concentration of phenolics was observed in many extract fractions (Table 2).

Available reports tend to show that secondary metabolites such as alkaloids, flavonoids, tannins, and other compounds of phenolic nature are responsible for the antimicrobial activities in higher plants [19]. Monoterpenes, sesquiterpenes, alcohols, and aldehydes have been reported to exhibit antibacterial activity in spices against respiratory tract infections. Cyclic terpene compounds have been reported to cause loss of membrane integrity and dissipation of proton motive force [19]. Therefore, presence of some of these phytochemicals along with phenolic compounds could to some extent justify the observed antibacterial activities in the present study. Many *T. cordifolia* extracts exhibited inhibition of pathogenic test bacteria (Table 3). The lower MBC values (1.29–22.73 mg/mL) against some of the bacteria indicated potential antimicrobial activity in the test plant.

The antimicrobial activities of phenolic compounds may involve multiple modes of action. Essential oils degrade the cell wall, interact with the composition and disrupt cytoplasmic membrane, damage membrane protein, interfere with membrane integrated enzymes, cause leakage of cellular components, coagulate cytoplasm, deplete the proton motive force, change fatty acid and phospholipid constituents, impair enzymatic mechanisms for energy production and metabolism, alter nutrient uptake and electron transport, influence the synthesis of DNA and RNA, and destroy protein translocation and the function of the mitochondrion in eukaryotes [28–30]. All of these mechanisms are not separate targets since some are affected as a consequence of another mechanism being targeted.

Extracts demonstrated considerable reducing power. The reductive capabilities of the plant extracts were compared with standard antioxidant ascorbic acid. However, the standard compound exhibited strong reducing power even at very low concentrations. In general, many test plant extracts demonstrated dose-dependent reducing power. Appreciable activity was found in BZ, CH, and EA extracts of *T. cordifolia *(Figure 1). Similar findings are also reported for other plant extracts [31]. The higher absorbance at 700 nm indicates higher reducing power in the extracts [32].

It has been reported that the reducing properties are generally associated with the presence of reductones, which have been shown to exert antioxidant action by breaking the free radical chain by donating a hydrogen atom [33]. Reductones are also reported to react with certain precursors of peroxide, thus preventing peroxide formation. The presence of reductants (antioxidants) in the herbal extracts causes the reduction of Fe^3+^/ferric cyanide complex to ferrous form [34]. It is therefore possible that activity of extracts might be due to the presence of higher amounts of reductones, which could react with free radicals to stabilise and block the radical chain reactions.

Polyphenolic contents of all the extracts appear to function as good electron and hydrogen atom donors and therefore should be able to terminate radical chain reaction by converting free radicals and ROS to more stable products. Higher activity observed in *T. cordifolia *extracts could also be attributed to the total phenolic contents.

The transition metal ion, Fe^2+^, possess the ability to move single electrons by virtue of which it can allow the formation and propagation of many radical reactions, even starting with relatively nonreactive radicals [31, 33]. The main strategy to avoid ROS generation that is associated with redox active metal catalysis involves chelating of the metal ions. Chelation therapy reduces iron-related complications and thereby improves quality of life and overall survival. Therefore, continuing search for finding alternative sources of iron chelating activity with lower side effects from plant sources bears significance.

Iron can stimulate lipid peroxidation by the Fenton reaction and also accelerates peroxidation by decomposing lipid hydroperoxides into peroxyl and alkoxyl radicals that can themselves abstract hydrogen and perpetuate the chain reaction of lipid peroxidation [33]. Ferrozine can quantitatively form complexes with Fe^2+^. In the presence of samples possessing chelating activity, the formation of red coloured complexes is decreased. Therefore, measurement of the rate of color reduction helps to estimate the chelating activity of the coexisting chelator present in the samples [31]. Our results have shown that the absorbance of coloured complex decreased linearly which indicated that the formation of Fe^2+^-ferrozine complex was not completed in the presence of *T. cordifolia *extracts, suggesting chelation of iron by phytochemicals present in this plant. Several reports on chelation of iron by other plant extracts also substantiate these findings [33, 35]. Phytochemicals present in* T. cordifolia* extracts interfered with the formation of ferrous-ferrozine complex, suggesting that they had chelating activity and captured ferrous ion before ferrozine.

It has been reported that chelating agents, which form *σ* bonds with a metal, are effective as secondary antioxidants because they reduce the redox potential, thereby stabilizing the oxidized form of the metal ion. Antioxidants inhibit interaction between metal and lipid through formation of insoluble metal complexes with ferrous ion [36]. The iron-chelating capacity test measures the ability of antioxidants to compete with ferrozine in chelating ferrous ion.

Remarkable progress has been made over the past two decades in understanding the molecular and cellular mechanisms of precancer and cancer progression. Nonetheless, the development of effective and safe agents for prevention and treatment of cancer remains slow, inefficient, and costly, with little to offer the high-risk population for primary cancer prevention and cancer survivors to prevent cancer recurrence. The key to effective chemotherapy and chemoprevention is the identification of chemotherapeutic and chemopreventive agents that can effectively inhibit cancer development without toxic side effects [36].

Many plant-derived compounds have been an important source of several clinically useful anticancer agents. These include vinblastine, vincristine, the camptothecin derivatives, topotecan and irinotecan, etoposide derived from epipodophyllotoxin, and paclitaxel [37]. Anticancer drugs having low side effects, inducing apoptosis and targeting specific cytotoxicity to the cancer cells, are drugs of choice. Keeping this in mind, we investigated the cytotoxic potential of extracts of *T. cordifolia* against human cancer cell lines.

Our results have shown that the phytochemicals present in *T. cordifolia* have potent cytotoxic and anticancer potential against MCF-7 cell line (Figure 3). Cancer cell lines used in the study exhibited differential sensitivity towards different plant extracts. The differential behaviour of cell lines may be due to different molecular characteristics of these cells. The present study clearly indicates that *T. cordifolia* extracts are very active against a few selected human cancer cell lines.

Polyphenols have been shown to possess antimutagenic and antimalignant effects. Moreover, flavonoids have a chemopreventive role in cancer through their effects on signal transduction in cell proliferation and angiogenesis [7]. The cytotoxic and antitumor properties of the extract may be due to the presence of these compounds. Adhvaryu et al. [14] have shown very high efficacy in *T. cordifolia* extracts against Dalton's lymphoma ascites (DLA) tumor model in Swiss Albino mice in terms of survival as well as tumor volume control. However, the exact mechanism is not clear. Available evidences suggest that DNA damage, inhibition of topoisomerase II, decline in clonogenicity and glutathione-S-transferase activity, activation of tumor associated macrophage, increase in lipid peroxidation, and LDH release to be probable mechanisms behind the cytotoxic activity [13]. The arabinogalactan present in aqueous extract of guduchi stem has also been shown to produce immunological activity. Many of the compounds mentioned above have been reported to be cytotoxic.

## 5. Conclusion

The study demonstrated the presence of various groups of phytochemicals in *T. cordifolia* extracts which are responsible for showing considerable antibacterial, antioxidant, and anticancer activities.

## Figures and Tables

**Table 1:** Phytochemical profile of *T. cordifolia* stem extracts.

Extracts	Phytochemicals									
	Reducing sugars	Anthraquinone	Terpenoids	Phenols	Flavonoids	Saponin	Tannin	Phlobatannin	Alkaloids	Cardiac glycosides
PE	−	+	+	+	−	−	−	−	+	−
BZ	−	+	+	+	+	−	−	−	−	−
CH	−	+	+	+	+	−	−	−	−	−
EA	+	+	+	+	+	−	+	−	−	−
AC	−	+	+	+	+	−	+	−	−	+
ET	−	+	+	+	+	−	+	−	+	+
AQ	−	+	+	+	+	+	+	−	−	−

Phytochemical analysis of *T. cordifolia* stem extracts was done as described in Section 2. PE: petroleum ether, BZ: benzene, CH: chloroform, EA: ethyl acetate, AC: acetone, ET: ethyl alcohol, and AQ: water; (+) present/detected; (−) not detected.

**Table 2:** Contents of total phenol in *T. cordifolia* stem extracts.

Extract fractions	Total phenol (mg, CE/g)
PE	13.75 ± 0.28
BZ	43.75 ± 0.05
CH	43.75 ± 0.05
EA	52.50 ± 0.02
AC	47.50 ± 0.13
ET	35.00 ± 0.02
AQ	8.75 ± 0.11

The values are represented as mg catechol equivalent per gram of sample (mg CE/g). The results are expressed as mean ± SEM (*n* = 3). PE: petroleum ether, BZ: benzene, CH: chloroform, EA: ethyl acetate, AC: acetone, ET: ethyl alcohol, and AQ: water.

**Table 3:** Antibacterial efficacy of *T. cordifolia* stem extracts against bacteria.

Extracts	*K*. *pneumoniae *	*Proteus* spp.	*E. coli *	*Pseudomonas* spp.
PE*	—	9.33 ± 0.58	—	8.33 ± 0.58
BZ^#^	—	10.33 ± 0.58	—	—
CH^#^	—	—	—	9.33 ± 0.58
EA	—	—	26.33 ± 0.58	17.67 ± 0.58
AC	11.33 ± 0.58	—	19.00 ± 1.00	14.67 ± 0.58
ET	12.33 ± 0.58	—	—	9.33 ± 0.58
AQ	10.33 ± 0.58	—	—	—
Antibiotics	18 ± 0.00 Imi	26 ± 0.00 Mero	37 ± 0.00 Mero	25 ± 0.00 Ptz

Zone of inhibition (ZOI) values are reported as mean ± SD of three replicates. Asterisks (*) and hash (^#^) represent extract contents in discs 5 mg/disc and 3.33 mg/disc, respectively. The extract contents present in other discs were 10 mg/disc. PE: petroleum ether, BZ: benzene, CH: chloroform, EA: ethyl acetate, AC: acetone, ET: ethyl alcohol, AQ: water, Imi: imipenem (10 *µ*g/disc), Mero: meropenem (10 *µ*g/disc), and Ptz: piperacillin tazobactam (100/10 *µ*g/disc).

**Table 4:** Minimum bactericidal concentration (MBC) of potential extracts derived from *T. cordifolia* stem.

Extract fractions	Bacteria			
	*K. pneumoniae *	*Proteus* spp.	*E. coli *	*Pseudomonas* spp.
PE	nt	7.1	nt	nt
BZ	nt	14.2	nt	nt
CH	nt	nt	nt	22.73
EA	nt	nt	4.21	1.29
AC	1.29	nt	4.21	4.21

MBC values are shown in mg/mL. The MBC values of potential extract fractions of *T. cordifolia* stem samples were determined as described in Section 2. Abbreviations: PE: petroleum ether, BZ: benzene, CH: chloroform, EA: ethyl acetate, AC: acetone and ET: ethyl alcohol fractions, nt: not tested.
